# The Role of Decompressive Craniectomy in the Context of Severe Traumatic Brain Injury: Summary of Results and Analysis of the Confidence Level of Conclusions From Systematic Reviews and Meta-Analyses

**DOI:** 10.3389/fneur.2019.01063

**Published:** 2019-10-10

**Authors:** Andrés M. Rubiano, Nancy Carney, Ahsan A. Khan, Mario Ammirati

**Affiliations:** ^1^INUB/MEDITECH Research Group, El Bosque University, Bogota, Colombia; ^2^NIHR Global Health Research Group on Neurotrauma, MEDITECH Foundation, Cali, Colombia; ^3^School of Medicine, Oregon Health & Science University, Portland, OR, United States; ^4^Center for Biotechnology, Department of Biology, College of Science and Technology, Temple University, Philadelphia, PA, United States; ^5^Innovative Neurotherapeutic Research Program Sbarro Health Organization, Temple University, Philadelphia, PA, United States

**Keywords:** brain injury, head trauma, decompressive craniectomy, ICP, TBI

## Abstract

**Introduction:** Traumatic brain injury (TBI) is a global epidemic. The incidence of TBI in low and middle-income countries (LMICs) is three times greater than in high-income countries (HICs). Decompressive craniectomy (DC) is a surgical procedure to reduce intracranial pressure (ICP) and prevent secondary injury. Multiple comparative studies, and several randomized controlled trials (RCTs) have been conducted to investigate the influence of DC for patients with severe TBI on outcomes such as mortality, ICP, neurological outcomes, and intensive care unit (ICU) and hospital length of stay. The results of these studies are inconsistent. Systematic reviews and meta-analyses have been conducted in an effort to aggregate the data from the individual studies, and perhaps derive reliable conclusions. The purpose of this project was to conduct a review of the reviews about the effectiveness of DC to improve outcomes.

**Methods:** We conducted a systematic search of the literature to identify reviews and meta-analyses that met our pre-determined criteria. We used the AMSTAR 2 instrument to assess the quality of each of the included reviews, and determine the level of confidence.

**Results:** Of 973 citations from the original search, five publications were included in our review. Four of them included meta-analyses. For mortality, three reviews found a positive effect of DC compared to medical management and two found no significant difference between groups. The four reviews that measured neurological outcome found no benefit of DC. The two reviews that assessed ICP both found DC to be beneficial in reducing ICP. DC demonstrated a significant reduction in ICU length of stay in the one study that measured it, and a significant reduction in hospital length of stay in the two studies that measured it. According to the AMSTAR 2 criteria, the five reviews ranged in levels of confidence from low to critically low.

**Conclusion:** Systematic reviews and meta-analyses are important approaches for aggregating information from multiple studies. Clinicians rely of these methods for concise interpretation of scientific literature. Standards for quality of systematic reviews and meta-analyses have been established to support the quality of the reviews being produced. In the case of DC, more attention must be paid to quality standards, in the generation of both individual studies and reviews.

## Introduction

Traumatic brain injury (TBI) remains one of the most serious public health problems worldwide, and in particular in low- and middle-income countries (LMICs) (1). Decompressive craniectomy (DC) has been used for the management of intracranial pressure (ICP) with severe TBI patients as a primary or prophylactic intervention, or as a secondary intervention when first-line therapies fail (2–4). Some studies in TBI populations have shown that DC improves ICP and cerebral perfusion pressure (CPP), contributing to improved long-term functional outcomes and reduction in costs (5–12). However, other studies show opposite results (13–15). Given the variation in results, leading to uncertainty about the actual benefit or not of the procedure, multiple systematic reviews and meta-analyses have been conducted to synthesize the results of the individual studies. However, in order to use the information from these reviews to make treatment and policy decisions, the findings must be critically considered within the context of the quality of the reviews.

Standards have been established for the assessment of the quality of systematic reviews and meta-analyses. One instrument that is widely used is AMSTAR 2 (A MeaSurement Tool to Assess systematic Reviews) (16). The instrument contains 16 individual domains, with 7 of them being “critical domains.” It was developed to provide health professionals and policy makers with a practical critical appraisal instrument to assess systematic reviews and meta-analyses that include randomized controlled trials (RCTs) as well as non-randomized studies (NRSs).

We conducted a literature search to identify systematic reviews and meta-analyses that compare the outcome for patients with severe TBI who receive DC with patients who receive standard medical management. We used the AMSTAR 2 instrument to assess the included publications. The purpose of this project was to summarize the findings of the publications in light of their AMSTAR 2 scores, and to identify potential improvements in the conduct of systematic reviews about DC that could contribute to the confidence in the findings. Thus, the emphasis in this paper is to critically assess the included systematic reviews/meta-analyses.

## Materials and Methods

The search included systematic reviews (SRs) and meta-analyses (MAs) published on the topic of DC in the treatment of severe TBI patients. A search strategy was developed including mesh terms and all field terms but also free text searches in search engines. The main strategy included: “brain injuries, traumatic”[MeSH Terms] OR “craniocerebral trauma”[MeSH Terms] AND (“decompressive craniectomy”[MeSH Terms] OR “decompressive craniectomy”[All Fields]) OR “decompressive craniotomy”[All Fields], filtering by study types of meta-analysis and systematic review (excluding all other types of studies). Systematic reviews/meta-analyses that included pathologies other than TBI, and those that focused on interventions other than DC specifically, were excluded.

Two investigators independently reviewed abstracts and full text articles. Discrepancies were resolved through consensus of three investigators.

## Results

### Literature Review

Nine hundred seventy three citations were obtained, most of which were not specific to the topic or did not meet the inclusion criteria. Six publications were retrieved that met the pre-determined inclusion criteria (17–22). We eliminated Sahuquillo (17) because it included only one study (23), which was included also in four of the other included reviews (18, 20–22). Thus, five SRs/MAs were included in this review.

The five reviews included 9 RCTs and 16 NRSs (see Table 1). Four of the five studies included both RCTs and NRSs, and one (21) included only RCTs. Three of the five studies used only RCTs in their MAs (18, 20, 21), one included both RCTs and NRSs in the MA (22), and one did not conduct a MA (19).

**Table 1:** Summary of systematic reviews/meta-analyses about decompressive craniectomy.

**Publication**	**Population and intervention**	**RCTs**	**Non-randomized studies**	**MA**	**Results**	**AMSTAR 2**
Wang et al. (18)	Early DC for severe TBI	(5, 14, 23)	(13, 24–27)	Yes for RCTs	Half the risk of death for DC group, but not statistically significant. ICP and hospital stay significantly lower for DC group.	Critically low. Violation of five and partial violation of one of the seven critical domains.
Barthélemy et al. (19)	DC and alternative means of decompression for severe TBI	(14, 28–30)	(31–38)	No	No difference in mortality or neurological outcomes between DC and medical management. Significantly better mortality and neurological outcomes for DC with multiple dural stabs compared to DC with open dural flap.	Low.Violation of two of the seven critical domains.
Zhang et al. (20)	DC for severe TBI	(5, 14, 23, 39)	(13, 24–27, 40)	Yes for RCTs	DC group had significantly lower mortality, ICP, and length of ICU and hospital stay than medical management group. DC group had significantly more complications. No significant difference in neurological outcomes between groups.	Low. Violation of one and partial violation of three of the seven critical domains.
Sahuquillo and Dennis (21)	DC for severe TBI	(14, 23, 41)	None included	Yes for RCTs	DC reduces the risk of mortality compared to medical management. DC does not reduce the risk of unfavorable outcomes.	Low. Violation of two and partial violation of two of the seven critical domains.
Fatima et al. (22)	Early DC for severe TBI	(14, 23, 41–43)	(26, 44)	Yes for all studies	Significantly lower risk of mortality with DC than with medical management ± late DC. No difference in neurological outcomes between early DC group compared to medical management ± late DC. Significantly lower risk of mortality with early DC than with late DC, but no difference in neurological outcomes.	Critically low. Violation of four and partial violation of one of the seven critical domains.

*RCT, randomized controlled trial; DC, decompressive craniectomy; ICP, intracranial pressure; MA, meta-analysis*.

### Assessment of Individual Reviews

The following summarizes each review with an emphasis on findings from the MA (when utilized) and RCTs, and presents the results of the AMSTAR 2 assessments.

Wang et al. (18) conducted a SR and MA to investigate the effect of early DC on mortality, ICP reduction, and hospital stay. They included three RCTs and five NRSs in their review, and used only the RCTs for the MA (see Table 1). For mortality, the pooled odds ratio (OR) was 0.531 [95% confidence interval (CI) 0.209–1.350, *Z* = 1.95, *p* = 0.183]. There was a significant reduction in ICP for the DC group compared to the non-DC group (pooled difference in means −2.081, 95% CI −2.796 to −1.366, *p* < 0.001). Also, the DC group had significantly fewer days in hospital than the non-DC group (pooled difference in means −9.907, 95% CI −16.250 to −3.565, *p* = 0.002). Thus, the findings from the pooled analysis indicate no significant effect of DC on mortality, and significantly reduced ICP and days in hospital.

Applying the AMSTAR 2 assessment criteria, the confidence in the findings from this review is critically low. They sustained violations in 5 of the 7 critical domains, and a partial violation for one additional critical domain (see Table 2).

**Table 2:** AMSTAR 2 individual domains and overall confidence scores for systematic reviews/meta analyses about decpompressive craniectomy.

	**Publications**				
**Review criteria**	**Fatima et al. (22)**	**Sahuquillo and Dennis (21)**	**Zhang et al. (20)**	**Barthélemy et al. (19)**	**Wang et al. (18)**
1. Did the research questions and inclusion criteria for the review include the components of PICO?	Yes	Yes	Yes	Yes	Yes
2. Did the report of the review contain an explicit statement that the review methods were established prior to the conduct of the review and did the report justify any significant deviations from the protocol?	No	Partial yes	No	No	No
3. Did the review authors explain their selection of the study designs for inclusion in the review?	Yes	Yes	Yes	Yes	Yes
4. Did the review authors use a comprehensive literature search strategy?	Partial yes	Yes	Yes	Yes	Partial yes
5. Did the review authors perform study selection in duplicate?	Yes	Yes	Yes	Yes	Yes
6. Did the review authors perform data extraction in duplicate?	No	No	Yes	No	No
7. Did the review authors provide a list of excluded studies and justify the exclusions?	No	No	Partial yes	No	No
8. Did the review authors describe the included studies in adequate detail?	Yes	Yes	Yes	Yes	Yes
9. Did the review authors use a satisfactory technique for assessing the risk of bias (RoB) in individual studies that were included in the review?	No	Partial yes	Yes	Yes	No
10. Did the review authors report on the sources of funding for the studies included in the review?	No	No	No	No	No
11. If meta-analysis was performed did the review authors use appropriate methods for statistical combination of results?	No	Yes	Yes	N/A	Yes
12. If meta-analysis was performed, did the review authors assess the potential impact of RoB in individual studies on the results of the meta-analysis or other evidence synthesis?	Yes	Yes	No	N/A	No
13. Did the review authors account for RoB in individual studies when interpreting/ discussing the results of the review?	Yes	Yes	Partial yes	Yes	No
14. Did the review authors provide a satisfactory explanation for, and discussion of, any heterogeneity observed in the results of the review?	Yes	No	Yes	Yes	Yes
15. If they performed quantitative synthesis did the review authors carry out an adequate investigation of publication bias (small study bias) and discuss its likely impact on the results of the review?	Yes	No	Partial yes	N/A	No
16. Did the review authors report any potential sources of conflict of interest, including any funding they received for conducting the review?	Yes	No	Partial yes	Partial yes	Partial yes
Overall confidence	Critically low	Low	Low	Low	Critically low

*Shaded rows indicate critical domains*.

*RoB, risk of bias*.

Barthelemy et al. (19) conducted a SR of studies that compared DC to medical management or to alternative means of surgical decompression, and reported on mortality, neurological outcomes measured by the Glasgow Outcome Scale (GOS), and ICP. The alternative means of decompression included craniotomy with controlled decompression and DC with multiple dural stabs (MDS). Four RCTs and eight NRSs were included in the review (see Table 1), which did not utilize a MA to combine data. Thus, the reported results and conclusions were derived from findings from the individual studies, rather than from pooled quantitative data. Among the RCTs, no significant benefits were found in mortality or neurological outcomes between the DC group and the medical management group, or between the DC group and the controlled decompressive craniectomy group. One study (28) found significantly lower mortality and higher function at discharge for patients who received MDS compared to DC. Of the two trials that reported on ICP, one (29) showed no benefit of DC and one (14) showed significant reduction in ICP with DC.

The AMSTAR 2 rating for this review is low confidence. There were violations in 2 of the 7 critical domains (see Table 2).

Zhang et al. (20) conducted a SR and MA to compare DC to medical management, and reported on mortality, neurological outcomes measured by the GOS, length of stay in the intensive care unit (ICU), length of stay in hospital, and complications. Of the ten included studies, four were RCTs and six were NRSs; the RCTs were included in the MA (see Table 1). For mortality, patients in the DC group had significantly lower risk of death compared to patients who received only medical management [Risk Ratio (RR) 0.59, 95% CI 0.47–0.74, *Z* = 4.60, *p* < 0.001]. Subgroup analysis showed a significant benefit for mortality with the early DC group (*p* < 0.001) but no difference for late DC (*p* = 0.89). For neurological outcomes, no significant difference was found between groups on the GOS or GOS-E (Extended GOS) at 6 months follow-up (RR 0.85, 95% CI 0.61–1.18, *Z* = 0.97, *p* = 0.33). However, the subgroup analysis of early DC showed a significant benefit in neurological outcome compared to late DC (RR 0.74, 95% CI 0.56–0.99, *Z* = 2.02, *p* = 0.04). Compared to medical management, DC significantly reduced ICP [mean difference (MD) −2.12 mmHg, 95% CI −2.81 to −1.43, *Z* = 6.03, *p* < 0.001]; significantly reduced length of ICU stay (MD −4.63 days, 95% CI −6.62 to −2.65, *Z* = 4.57, *p* < 0.001); and significantly reduced length of stay in hospital (MD −14.39 days, 95% CI −26.00 to −2.78, *Z* = 2.43, *p* = 0.02). The DC group sustained significantly more complications than the medical management group (RR 1.94, 95% CI 1.31–2.87, *Z* = 3.33, *p* = 0.0009). In sum, the DC group had significantly lower mortality, ICP, and length of ICU and hospital stay than the medical management group, and had significantly more complications. There was no difference between groups in neurological outcomes.

The AMSTAR 2 rating for this review is low confidence. There was a violation of 1 of the 7 critical domains, and partial violations of 3 critical domains.

Sahuquillo and Dennis (21) limited their SR and MA to only RCTs comparing DC to medical management. They reported on mortality and neurological outcomes measured by the GOS-E. Three trials were included in the review. Pooled results indicated significantly lower mortality for the DC group compared to the medical management group (RR 0.61, 95% CI 0.48–0.78, *I*^2^ = 38%). There was no significant difference between groups in neurological outcome measured at 6 months follow-up (RR 1.08, 95% CI 0.93–1.20, *I*^2^ = 78%). Authors reported DC was superior to medical management in reducing ICP, but did not provide quantitative data. To summarize, this review found that DC reduces the risk of mortality compared to medical management, reduces ICP, and does not reduce the risk of unfavorable neurological outcomes.

The AMSTAR 2 rating for this review is low confidence. There was violation of 2 of the 7 critical domains, and partial violation of 2 of the critical domains.

Fatima et al. (22) conducted a SR and MA to compare outcomes from early DC with those from medical management with or without (±) late DC. They reported on mortality and neurological outcomes measured by the GOS. Of seven included reviews, five were RCTs and two were NRSs (see Table 1). All studies were included in the MA. There was significantly lower mortality for the early DC group compared to the medical management ± late DC group (RR 0.62, 95% CI 0.40–0.94, *p* = 0.03). There was no difference between groups for neurological outcomes (OR 1.00, 95% CI 0.75–1.34, *p* = 0.99). A subgroup analysis indicated a significant reduction in mortality for the early DC group compared to the late DC group (RR 0.43, 95% CI 0.26–0.71, *p* = 0.0009), but no difference in neurological outcomes (OR 1.30, 95% CI 0.75–2.27, *p* = 0.35). In sum, when early DC is compared to medical management ± late DC, there is a significantly lower risk of mortality with early DC but no difference in neurological outcomes; the findings are the same in subgroup analysis that compares early DC to late DC.

The AMSTAR 2 rating for this review is critically low. They sustained violations in 4 of the 7 critical domains and a partial violation in 1 of the critical domains.

### Summary of the Findings From the Five Reviews

For mortality, three reviews found a positive effect of DC compared to medical management and two found no significant difference between groups. The four reviews that measured neurological outcome found no benefit of DC. The two reviews that assessed ICP both found DC to be beneficial in reducing ICP. DC demonstrated a significant reduction in ICU length of stay in the one study that measured it, and a significant reduction in hospital length of stay in the two studies that measured it.

Subgroup analyses showed the following: early DC reduced mortality compared to late DC, but did not improve neurological outcomes in one study; in another study, DC was associated with significantly more complications; in a third study that assessed alternative means of decompression, dural stabs improved mortality and neurological function compared to open dural flap.

### Summary of the Quality of the Reviews Based on AMSTAR 2

The scoring system for the AMSTAR 2 instrument is in Table 3. As stated earlier, there are 16 domains that constitute the instrument, with 7 designated as “critical domains.” The shaded columns in Table 2 are the critical domains for the instrument.

**Table 3:** AMSTAR 2 scoring system.

**High–**Zero or one non-critical weakness: The systematic review provides an accurate and comprehensive summary of the results of the available studies that address the question of interest
**Moderate–**More than one non-critical weakness^*^: The systematic review has more than one weakness, but no critical flaws. It may provide an accurate summary of the results of the available studies that were included in the review
**Low–**One critical flaw with or without non-critical weaknesses: The review has a critical flaw and may not provide an accurate and comprehensive summary of the available studies that address the question of interest
**Critically low–**More than one critical flaw with or without non-critical weaknesses: The review has more than one critical flaw and should not be relied on to provide an accurate and comprehensive summary of the available studies

* *Multiple non-critical weaknesses may diminish confidence in the review and it may be appropriate to move the overall appraisal down from moderate to low confidence*.

According to the AMSTAR 2 criteria, the five reviews ranged in levels of confidence from low to critically low. The most common violations were in critical domain #2, “Did the report of the review contain an explicit statement that the review methods were established prior to the conduct of the review and did the report justify any significant deviations from the protocol?” and in critical domain #7, “Did the review authors provide a list of excluded studies and justify the exclusions?” None of the reviews adhered completely to these criteria. Other violations include inadequate investigation of publication bias (domain 15) and insufficient technique for assessing risk of bias (domain 9). In light of the AMSTAR 2 scores for these reviews, confidence in the reported findings is low.

## Discussion

As stated in the Introduction, the purpose of this project was to summarize the findings from SRs and MAs about the effectiveness of DC to improve outcomes for patients with severe TBI, and to consider those findings in the context of their AMSTAR 2 scores. In general, the reviews report that DC can decrease mortality, reduce ICP, and minimize days in the ICU and hospital, but does not serve to improve neurological function. However, based solely on the AMSTAR 2 criteria, we report a low level of confidence in these findings. They are in part, however, consistent with findings from Class 1 RCTs (14, 41). These RCTs, as well as other literature about DC, have been the focus of intense and ongoing critical conversation (39, 45, 46), and have inspired the gathering of a formidable group of clinical experts who generated a consensus statement about DC (39).

DC is a complex and multi-faceted intervention. A key flaw in DC studies and reviews has been a lack of sufficient attention to this complexity in the conduct the studies and the analyses. Cranial decompression is a procedure with several technical variations (primary vs. secondary, early vs. delayed, bifrontal vs. unilateral). Furthermore, timing of the DC is a source of heterogeneity within and across studies. The SRs and MAs mixed these variations in the DC intervention in pooled analyses.

The findings for the effect of DC on mortality from the five SRs/MAs included in this review were mixed; three found a positive effect and two found no difference between groups. However, all four SRs/MAs that measured neurological outcomes concluded no benefit from DC. To consider this finding, we focus on the factor of the timing of the DC procedure from the two Class 1 trials included in this review—DECRA (14) and RESCUEicp (41). Both trials aimed to treat patients with refractory elevated intracranial pressure. The median time from injury to surgery in the DECRA trial was 38.1 h [interquartile range (IQR) 27.1–55.0]. Timing for the RESCUEicp trial was reported as follows: time from injury to initial treatment: <12 h. *N* = 120, >12 h. *N* = 76; median time from initial treatment to randomization 44.3 h (IQR 16.8–80.9); median time from randomization to surgery 2.2 h IQR 1.3–5.1, mean 7.5 h (95% CI 5–9.9). Thus, the timing of the DC procedure in these trials ranged from hours to days, being technically studies of secondary DC.

Some neurosurgeons believe that DC is best performed as a last ditch procedure, as it is drastic and it has a high complication rate. However, in the setting of potentially intractable ICP, perhaps the delay in timing—meant to be a conservative approach—is at least in part a source of the observed poor outcomes. Are poor outcomes an inevitable result of delayed surgery, and overly conservative surgical approaches? To date, a trial of early DC with a pre-specified, controlled surgical approach has not been conducted. Such a trial could run the risk of over-aggressive use of DC. The next step might be a systematic review and report of the evidence for patient and injury characteristics that are indicators of the need for immediate surgery; then a trial randomizing this subset of patients to DC or medical management.

Timing is only one factor that varies across studies, and is used here as an example of the possible sources of study and SR/MA heterogeneity.

## Conclusion

Systematic reviews and meta-analyses are important approaches for aggregating information from multiple studies but are susceptible to misinterpretation of the results due to methodological flaws. Clinicians rely on these methods for concise interpretation of scientific literature. Standards for assessing SRs and MAs have been established to support the quality of the reviews being produced. In the case of DC, more attention must be paid to quality standards, in the analysis of both individual studies and reviews. In the included reviews, the procedure was found to decrease mortality, reduce ICP, and minimize days in the ICU and hospital, but was not found to improve neurological function. However, according to the assessment of the reviews utilizing a validated instrument, these conclusions have a low level of confidence.

## Author Contributions

AR, NC, AK, and MA contributed equally to the conception, writing, and preparation of the manuscript.

### Conflict of Interest

The authors declare that the research was conducted in the absence of any commercial or financial relationships that could be construed as a potential conflict of interest.
